# Characterization and clinical enrichment of HLA-C*07:02-restricted Cytomegalovirus-specific CD8^+^ T cells

**DOI:** 10.1371/journal.pone.0193554

**Published:** 2018-02-28

**Authors:** Fabian Schlott, Dominik Steubl, Stefanie Ameres, Andreas Moosmann, Stefan Dreher, Uwe Heemann, Volker Hösel, Dirk H. Busch, Michael Neuenhahn

**Affiliations:** 1 Institute for Medical Microbiology, Immunology and Hygiene, Technische Universität München, Munich, Germany; 2 DZIF—National Centre for Infection Research, Munich, Germany; 3 Department of Nephrology, Klinikum rechts der Isar, Technische Universität München, Munich, Germany; 4 DZIF Research Group "Host Control of Viral Latency and Reactivation" (HOCOVLAR), Research Unit Gene Vectors, Helmholtz Zentrum München, Munich, Germany; 5 Technical University Munich, Chair of Biomathematics, Garching, Germany; University of St Andrews, UNITED KINGDOM

## Abstract

Human Cytomegalovirus (CMV) reactivation remains a major source of morbidity in patients after solid organ and hematopoietic stem cell transplantation (HSCT). Adoptive T cell therapy (ACT) with CMV-specific T cells is a promising therapeutic approach for HSCT recipients, but might be counteracted by CMV’s immune evasion strategies. HLA-C*07:02 is less susceptible to viral immune evasion suggesting HLA-C*07:02-restricted viral epitopes as promising targets for ACT. For a better understanding of HLA-C*07:02-restricted CMV-specific T cells we used recently generated reversible HLA-C*07:02/IE-1 multimers (Streptamers) recognizing a CMV-derived Immediate-Early-1 (IE-1) epitope and analyzed phenotypic and functional T cell characteristics. Initially, we detected very high frequencies of HLA-C*07:02/IE-1 multimer^+^ T cells (median = 11.35%), as well as robust functional responses after stimulation with IE-1 peptide (IFNγ^+^; median = 5.02%) in healthy individuals. However, MHC-multimer^+^ and IFNγ-secreting T cell frequencies showed a relatively weak correlation (r^2^ = 0.77), which could be attributed to an unexpected contribution of CMV-epitope-independent KIR2DL2/3-binding of HLA-C*07:02/IE-1 multimers. Therefore, we developed a MHC-multimer double-staining approach against a cancer epitope-specific HLA-C*07:02 multimer to identify truly HLA-C*07:02/IE-1 epitope-specific T cells. The frequencies of these truly HLA-C*07:02/IE-1 multimer^+^ T cells were still high (median = 6.86%) and correlated now strongly (r^2^ = 0.96) with IFNγ-secretion. Interestingly, HLA-C*07:02/IE-1-restricted T cells contain substantial numbers with a central memory T cell phenotype, indicating high expansion potential e.g. for ACT. In line with that, we developed a clinical enrichment protocol avoiding epitope-independent KIR-binding to make HLA-C*07:02/IE-1-restricted T cells available for ACT. Initial depletion of KIR-expressing CD8^+^ T cells followed by HLA-C*07:02/IE-1 Streptamer positive selection using paramagnetic labeling techniques allowed to enrich successfully HLA-C*07:02/IE-1-restricted T cells. Such specifically enriched populations of functional HLA-C*07:02/IE-1-restricted T cells with significant central memory T cell content could become a potent source for ACT.

## Introduction

Human Cytomegalovirus (CMV), a β-herpesvirus, causes lifelong latent infections in humans, reaching a seroprevalence of 50–90% [1, 2]. CMV-infection of immunocompetent individuals takes usually a subclinical course, but reactivation or primary infection in immunocompromised patients after solid organ transplantation (SOT) or hematopoietic stem cell transplantation (HSCT) can lead to severe morbidity and mortality [3, 4]. The introduction of potent antiviral agents reduced the incidence of CMV manifestations, but these drugs are associated with limiting side effects like interstitial transplant fibrosis in kidney transplant recipients [5] and bone marrow suppression after HSCT [6]. As CMV-specific cytotoxic T cells play an essential role in viral control [7, 8], alternative strategies such as adoptive transfer of CMV-specific T cells have been intensively investigated during the last years [9–13]. Interestingly, selection of early-differentiated memory T cells could be advantageous for sustained reconstitution in HSCT patients, in particular if applied prophylactically [14, 15]. Additionally, it was shown that the use of reversible MHC Streptamers enables clinical purification of minimally manipulated CMV-specific T cells to high purity, avoiding complex regulatory requirements for advanced therapy medicinal products (ATMPs) [16–19]. Finally, T cell responses mediated by HLA-I molecules not belonging to HLA-A and–B alleles could play an important role in viral control. One promising CMV epitope is the immediate early-1 (IE-1) peptide_309-317_, which is restricted to HLA-C*07:02 [20]. HLA-C*07:02 is an inhibitory ligand for Killer-cell immunoglobulin-like receptor (KIR) 2DL2/3, which inhibits Natural Killer (NK) cell-mediated killing [20–22]. Interestingly, HLA-C*07:02 is much less susceptible to viral immune evasive strategies than CMV epitope-presenting HLA-A or–B molecules [20], presumably to avoid NK cell cytotoxicity. Furthermore, a possible evolutionarily beneficial inheritance from the Neandertalian genome [23] and its high allelic frequency of approximately 15% within the Caucasian population [24] hint both to a putative selection advantage of HLA-C*07:02 [25]. This makes the HLA-C*07:02-presented CMV epitope IE-1_309−317_ also an interesting new target for vaccination, adoptive T cell therapy and immune monitoring. In order to learn more about its role within CMV-specific immunity, we developed suitable MHC multimer staining protocols, intracellular cytokine staining (ICS) and magnetic serial enrichment with recently generated reversible MHC multimers [19, 20]. Our results demonstrate that HLA-C*07:02-restricted IE-1_309−317_ CD8^+^ T cell responses are detectable with high frequencies in CMV-seropositive donors and have some characteristic differentiation patterns. Depletion of T cell subsets mediating epitope-independent KIR-binding allowed to develop a novel enrichment protocol for robust and highly efficient enrichment of HLA-C*07:02-restricted CMV IE-1-specific T cell populations.

## Materials and methods

### Human material

Cells were purified from whole blood of healthy donors (n = 20). Written informed consent was obtained from all individuals and the collection was approved by the local Institutional Review Board according to national law (Ethics committee of the Faculty of Medicine, Technical University Munich (55/14)) covering the presented work reported in this manuscript. In addition, between 2011 and 2014, we monitored patients who received a kidney transplant at the University Hospital Klinikum rechts der Isar, Munich, Germany in an observational trial. This study was also approved by the Ethics committee of the Faculty of Medicine, Technical University Munich (5055/11) and (5495/12) and was in accordance with the declaration of Helsinki and the declaration of Istanbul. All enrolled patients gave their written informed consent approving the presented work reported in this manuscript. Here, one exemplary HLA-typed patient was used for characterization of HLA-C*07:02/IE-1-restricted CMV-specific CD8^+^T cells.

### Isolation and cryopreservation of peripheral blood mononuclear cells (PBMC)

For PBMC isolation, heparinized blood was diluted 1:1 with phosphate-buffered saline (PBS, Biochrom AG, Berlin, Germany) and purified using Ficoll (Biocoll, Biochrom AG, Berlin, Germany) differential centrifugation at 700g for 25 minutes. After separation, PBMCs were washed twice with PBS, respectively RPMI (Sigma-Aldrich, Taufkirchen, Germany). 90% fetal calf serum (FCS, Biochrom AG) and 10% dimethyl sulfoxide (DMSO, Sigma-Aldrich) solution were used for cryopreservation of PBMCs in liquid nitrogen.

### MHC Streptamers

Generation of MHC Streptamers was performed as previously described [19, 26]. In brief, inclusion body-derived recombinant MHC heavy chain was urea-denatured and refolded in the presence of peptide and β2 microglobulin. Complexes were purified by fast protein liquid chromatography (FPLC). Multimerization was performed with Strep-Tactin^®^ PE (cell staining; IBA Lifesciences, Goettingen, Germany), Strep-Tactin^®^ APC (cell staining; IBA Lifesciences) or Strep-Tactin^®^ magnetic beads (cell isolation; IBA Lifesciences). The following MHC Streptamers were used: HLA-B*07:02/pp65_417-427_ (TPRVTGGGAM), HLA-C*07:02/IE-1_309−317_ (CRVLCCYVL) and HLA-C*07:02/MAGE-A12_170-178_ (VRIGHLYIL).

### MHC Streptamer staining

MHC Streptamers (0.4μg/1x10^6^ cells) were freshly multimerized on 4°C for 45min with Strep-Tactin^®^ PE or APC (0.15μg/1x10^6^ cells). Cryopreserved PBMC were thawed and incubated for 10 minutes on ice with 2 μg/ml ethidium bromide monoazide (EMA, Sigma-Aldrich) for live/dead discrimination. Phenotypical marker staining by anti-CCR7 FITC (R&D Systems, USA) was performed at 37°C for 20 minutes. All other steps were performed at 4°C. For MHC Streptamer double staining, cells were first stained with CMV-unspecific (MAGE-A12_170-178_) MHC Streptamer conjugated with PE for 50 minutes, followed by two washing steps. Thereafter, PBMCs were stained with CMV-specific (IE-1_309−317_) MHC Streptamer conjugated with APC for 20 minutes. If single staining was performed the first MHC staining step was skipped. After MHC staining, cells were incubated for 30 minutes at 4°C with anti-CD3 eFluor 450 (eBioscience, San Diego, USA), anti-CD8 PerCP (BD Biosciences, Heidelberg, Germany), anti-CD19 ECD (Beckman Coulter, Krefeld, Germany) and anti CD45RO PC7 (eBioscience). Optional: For KIR visualization, MHC staining was followed by a staining for 30 minutes at 4°C with anti-KIR2DL2/3 FITC (CD158b, BD Bioscience), anti-CD3 eFluor 450 (eBioscience), anti-CD8 PerCP (BD Biosciences) and anti-CD19 ECD (Beckman Coulter). In either case, cells were fixed with 2% paraformaldehyde (PFA, Sigma-Aldrich) and acquired using a BD^TM^ LSR II (BD Biosciences) and analyzed by FlowJo software (FlowJo LLC, Ashland, USA). The gating strategy is shown in S1A Fig.

### Magnetic cell separation

The depletion of KIR2DL2/3^+^ cells was achieved using a PE-labeled KIR2DL2/3 antibody (CD158b, BD Bioscience), anti-PE MicroBeads (Miltenyi Biotec GmbH, Bergisch Gladbach, Germany) and LD Columns (Miltenyi Biotec GmbH) according to the Miltenyi protocol. Enrichment of HLA-C*0702/IE-1-specific CD8^+^ T cells was performed using Strep-Tactin® Magnetic Microbeads (IBA Lifesciences) for 20 minutes at 4°C and the StrepMan Magnet for 15 ml and 50 ml tubes (IBA Lifesciences) followed by a 10 minutes incubation at room temperature with 1mM D-(+)-Biotin (IBA Lifesciences).

### Intracellular cytokine staining (ICS)

After thawing of PBMC, cells were rested for 18h (2x10^6^ cells/ml RPMI) at 37°C/5% CO_2_ and stimulated *ex vivo* with 2μg/ml of peptide (IBA Lifesciences, Germany) and 1μl/ml co-stimulatory antibodies CD28 and CD49d (both BD Bioscience) for 4.5 h at 37°C/ 5% CO_2_. After 1 h 0.01 μg/μl Brefeldin A (Sigma-Aldrich) was added. Life/dead discrimination was achieved with 2 μg/ml EMA for 10 minutes on ice. Surface staining was performed with anti-CD8 PerCP, anti-CD3 eFluor 450 and anti-CD19 ECD for 30 minutes at 4°C and afterwards cells were permeabilized/fixed for 20 minutes on ice using the BD^TM^ Cytofix/Cytoperm kit (BD Biosciences). For intracellular staining PBMCs were incubated with anti-IFNγ Alexa Fluor® 700 (eBioscience), anti-TNFα PC7 (eBioscience), anti-IL-2 APC (BD Bioscience) and anti-GM-CSF PE (BD Bioscience) for 30 minutes on ice. Cells were acquired using a BD^TM^ LSR II (BD Biosciences) and analyzed by FlowJo software (FlowJo, USA). The gating strategy is shown in S1B Fig.

### Quantification of absolute cell counts and statistical analysis

For the calculation of absolute CMV-specific T cells, the BD^TM^ Trucount kit (BD Biosciences) was used. Statistical analysis were performed with the Mann-Whitney *U* test and calculated by GraphPad Prism 5 (GraphPad Software, La Jolla, USA) for Windows.

## Results

### Detection of HLA-C*07:02/IE-1 multimer-binding CD8^+^ T cell populations in CMV seropositive donors

**Fig 1:**
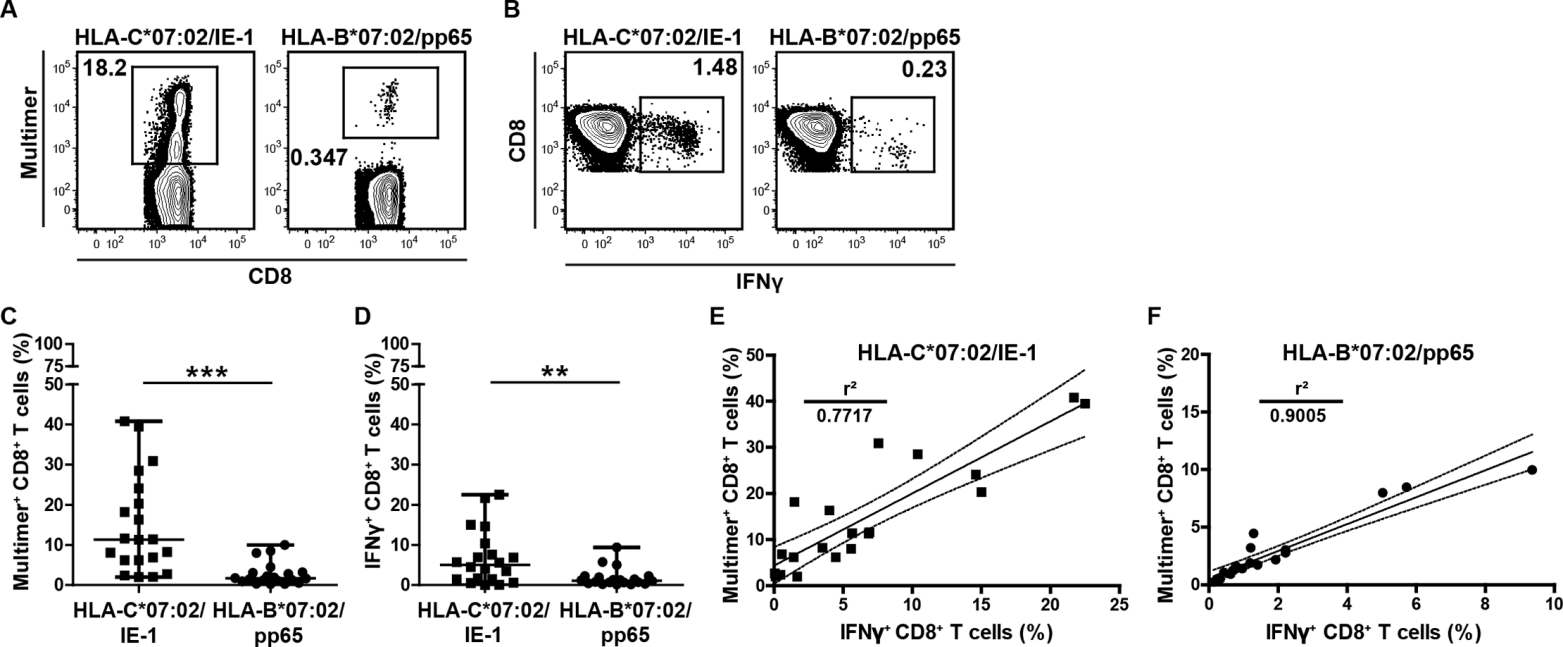
Intraindividual comparison of HLA-C*07:02/IE-1- and HLA-B*07:02/pp65-restricted CMV-specific T cells. Representative **(A)** multimer and **(B)** intracellular cytokine staining of CD8^+^ T cells from a healthy donor with specificity for the indicated CMV epitopes. For intracellular cytokine staining, cells were restimulated with the corresponding epitopes. **(C-F)** Comparative T cell analysis of a group of healthy donors (n = 20) carrying both CMV-specific T cell populations. **(C)** Multimer staining of CD8^+^ T cells for the corresponding epitopes. **(D)** ICS of CD8^+^ IFNγ-producing T cells after stimulation with corresponding epitopes. **(E, F)** Regression analysis of multimer^+^ and IFNγ^+^ CD8^+^ T cell frequencies with either HLA-C*07:02/IE-1 or HLA-B*07:02/pp65 specificity. Plots were uniformly pre-gated on living CD3^+^/CD8^+^ lymphocytes. Statistical analyses were performed with the Mann-Whitney *U* test. * = p < 0.05, ** = p < 0.01, *** = p < 0.001.

We used recently generated MHC multimers [20] to determine the distribution of HLA-C*07:02/IE-1-restricted CMV-specific T cells (nonameric IE-1-derived peptide CRVLCCYVL; amino acids 309–317) in HLA-C*07:02^+^ volunteers by HLA-C multimer staining. Due to a known linkage disequilibrium, HLA-C*07:02 is nearly always accompanied in Caucasians by HLA-B*07:02 [24], which is known to present an immunodominant epitope of the CMV tegument protein pp65 (nonameric pp65-derived peptide TPRVTGGGAM; amino acids 417–427) [27]. Therefore, we could compare HLA-C*07:02/IE-1 and HLA-B*07:02/pp65 multimer^+^ PBMC intraindividually. As exemplified in Fig 1A, HLA-C*07:02/IE-1 multimer^+^ CD8^+^ T cells frequencies partially reached enormous sizes and were considerably larger than HLA-B*07:02/pp65 multimer^+^ cell populations (18.2% vs 0.347%). In order to confirm the specificity of the MHC multimer staining, we performed within the same donor ICS after PBMC stimulation with the corresponding IE-1 and pp65 peptides. We detected 1.48% (HLA-C*07:02/IE-1) and 0.23% (HLA-B*07:02/pp65) Interferon-γ (IFN-γ)-producing CD8^+^ T cells (Fig 1B) revealing a clear discrepancy between HLA-C*07:02/IE-1 multimer staining and ICS; in contrast, for the B*07:02/pp65 epitope we found a good match between HLA multimer staining and ICS. High HLA-C*07:02/IE-1 multimer frequencies in combination with substantially lower ICS frequencies could be confirmed by the analysis of twenty HLA-C*07:02^+^ autologous blood donors. We found two digit frequencies of HLA-C*07:02/IE-1 multimer^+^ CD8^+^ T cells (median = 11.35%; 1.96–40.8%), but significantly lower HLA-B*07:02/pp65 multimer^+^ T cell frequencies (median = 1.67%; 0.239–9.98%; p < 0.001; Fig 1C). In ICS, we detected median IFNγ^+^ CD8^+^ T frequencies of 5.02% (0.04–22.5%) for HLA-C*07:02/IE-1 and 1.07% (0.13–9.36%) for HLA-B*07:02/pp65 (p = 0. 0098; Fig 1D). Similar observations were made for TNFα and GM-CSF (S2A and S2C Fig). Furthermore, we have analyzed in a subgroup of donors (n = 6) the production of CD107a, a marker of CD8^+^ T cell degranulation. We detected median CD107a^+^ CD8^+^ T frequencies of 2.74% (0.46–12.4%) for HLA-C*07:02/IE-1 and 0.57% (0.18–1.56%) for HLA-B*07:02/pp65 (p = 0. 0649; S3 Fig). Taken together, HLA-C*07:02/IE-1 CD8^+^ T cell frequencies were found to be significantly higher than T cell frequencies restricted to the well-known immunodominant CMV epitope HLA-B*07:02/pp65, both in MHC multimer and ICS staining. However, median HLA-C*07:02/IE-1 CD8^+^ T cell frequencies measured by ICS were substantially lower than in HLA-C multimer staining. Correlation of MHC multimer^+^ and IFNγ^+^ CD8^+^ T cells revealed only a moderate fit of r^2^ = 0.77 for HLA-C*07:02/IE-1 (Fig 1E), but a very good fit of r^2^ = 0.90 for HLA-B*07:02/pp65-restricted CD8^+^ T cells (Fig 1F). The comparatively low HLA-C multimer/ICS correlation indicated that in contrast to the long experiences with CMV-specific HLA-A and HLA-B multimers, perhaps additional receptors might be able to bind to HLA C*07:02 multimers in an epitope-independent manner.

### T cell receptor-independent binding of HLA-C-multimers by KIR-expressing CD8^+^ T cells

**Fig 2:**
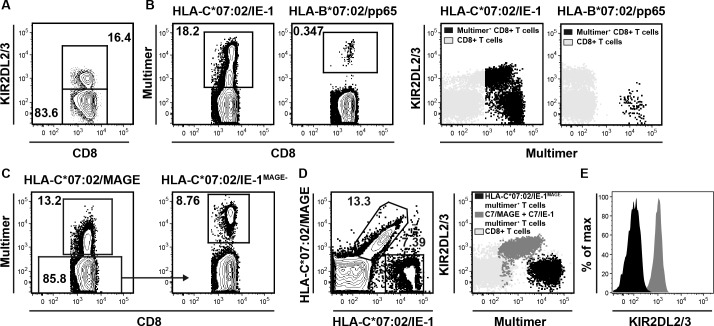
KIR-expressing CD8^+^ T cells bind HLA-C-multimers epitope-independently. **(A)** Exemplary depiction of KIR-expression on CD8^+^ T cells by KIR2DL2/3 staining. **(B)** Multimer staining of CD8^+^ T cells for the indicated epitopes and overlay of multimer^+^ CD8^+^ T cells (blue) and KIR2DL2/3 staining (grey). **(C)** Gating-strategy to exclude KIR-associated epitope-independent binding by multimer double staining with irrelevant tumor-epitope HLA-C*07:02/MAGE and HLA-C*07:02/IE-1 multimers. **(D)** Multimer double staining and overlay with KIR2DL2/3 of double- (red) and single-multimer^+^ (HLA-C*07:02/IE-1^MAGE-^, blue) CD8^+^ T cells. **(E)** Histogram for KIR2DL2/3-staining of double- (red) and single-multimer^+^ (blue) CD8^+^ T cells. T cells were uniformly pre-gated on living CD3^+^/ CD8^+^ lymphocytes.

The receptor KIR2DL2/3, which is widely expressed on NK cells, is known to inhibit their function by binding to HLA-C group 1 molecules including HLA-C*07:02. [28]. Due to the discrepancy between HLA-C*07:02/IE-1 multimer^+^ and IFNγ^+^ CD8^+^ T cell frequencies, we analyzed the KIR-expression on CD8^+^ T cells. As described already in the literature [29], we could detect a substantial fraction (16.4%) of KIR2DL2/3-expressing CD8^+^ T cells (Fig 2A). Further on, we analyzed HLA-C*07:02/IE-1 multimer^+^ CD8^+^ T cells from the donor in Fig 1A for KIR2DL2/3 receptor expression. This analysis revealed that roughly 50% of the HLA-C*07:02-multimer^+^ T cells were KIR2DL2/3 positive (Fig 2B), most of them showing lower MHC multimer staining mean fluorescence intensity (MFI). In contrast, we could detect hardly any KIR2DL2/3-expressing HLA-B*07:02/pp65 multimer^+^ cells (Fig 2B). In order to establish a strategy that allows to distinguish TCR-independent HLA-C staining from epitope-specific HLA-C*07:02 multimer staining, we generated a second HLA-C*07:02-restricted multimer, refolded with a melanoma antigen (nonameric MAGE-A12-derived peptide VRIGHLYIL; amino acids 170–178) and developed a HLA-C multimer double staining protocol. And indeed, excluding HLA-C*07:02/MAGE multimer^+^ cells enables to visualize single-positive, KIR-adjusted truly HLA-C*07:02/IE-1-specific CD8^+^ T cells indicated as HLA-C*07:02/IE-1^MAGE-^ (Fig 2C). Analyzing the content of KIR2DL2/3^+^ cells within the different HLA-C multimer^+^ populations, we confirmed that all MHC multimer double-positive cells were KIR2DL2/3 positive (Fig 2D and 2E, red), whereas single HLA-C*07:02/IE-1 multimer^+^ cells were KIR2DL2/3 negative (Fig 2D and 2E, blue). Taken together, our newly established HLA-C multimer double-staining approach allows the exclusion of epitope-unspecifically binding (mainly KIR2DL2/3-expressing) cells and the correct identification of HLA-C*07:02/IE-1-specific T cells.

### Distribution of KIR-adjusted HLA-C*07:02/IE-1^MAGE-^ multimer^+^ T cells in healthy donors

**Fig 3:**
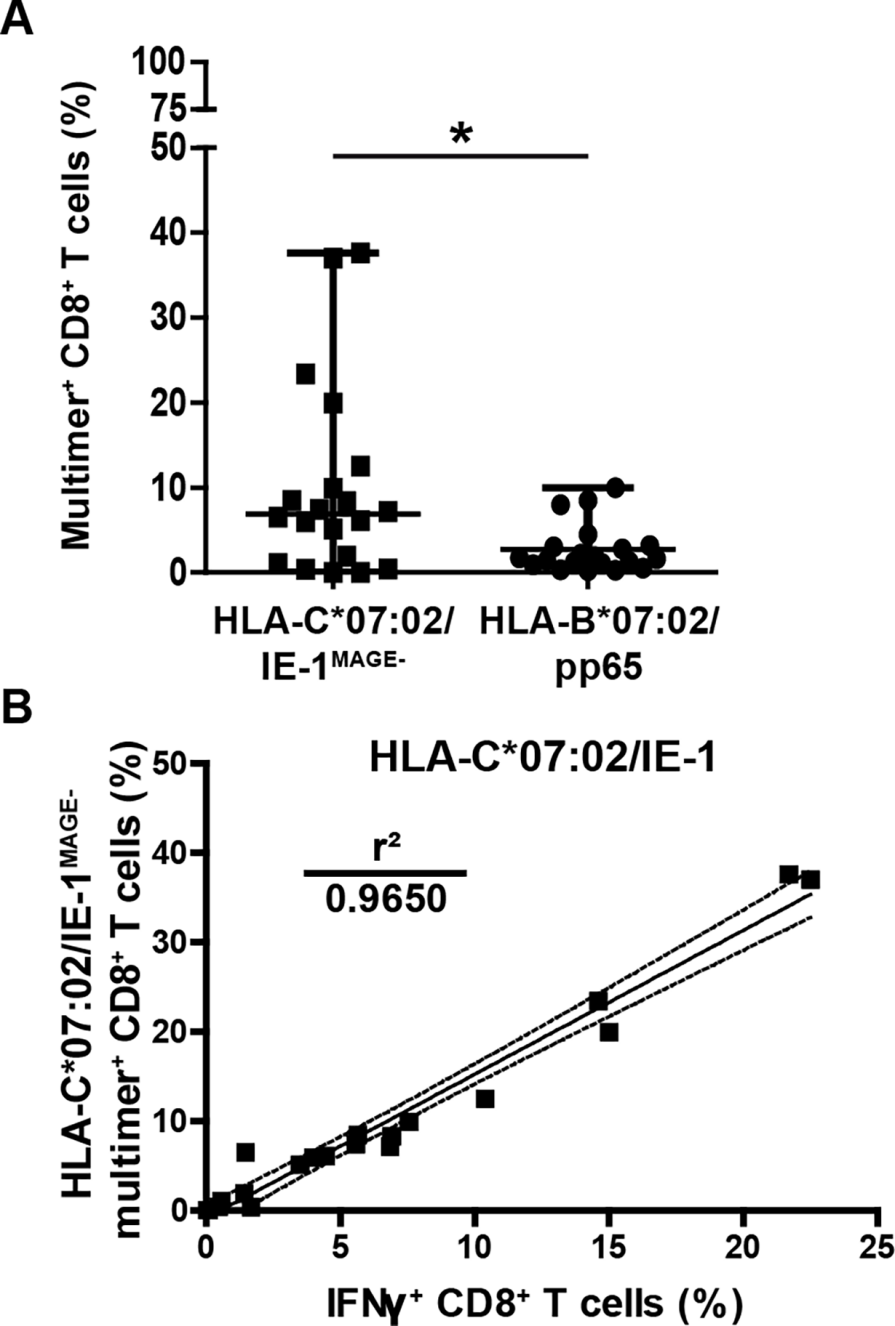
Reanalysis of KIR-adjusted HLA-C*07:02/IE-1-specific T cell frequencies. HLA-C*07:02/IE-1 multimer^+^ CD8^+^ T cell frequencies for the donors from Fig 1 were adjusted for epitope-independent KIR-binding by excluding cells with HLA-C*07:02/MAGE multimer co-staining. **(A)** Multimer stainings of HLA-C*07:02/IE-1^MAGE-^- and HLA-B*07:02/pp65-specific CD8^+^ T cells are compared. **(B)** Regression analysis of HLA-C*07:02/IE-1^MAGE-^ multimer^+^ and IFNγ^+^ HLA-C*07:02/IE-1 peptide-stimulated CD8^+^ T cells. T cells were uniformly pre-gated on living CD3^+^/CD8^+^ lymphocytes. Statistical analysis was performed with the Mann-Whitney *U* test. * = p < 0.05, ** = p < 0.01, *** = p < 0.001.

We used PBMCs from the donors out of Fig 1 and performed HLA-C multimer double-staining to exclude TCR-independent KIR2DL2/3 binding. We found a median frequency of 6.86% (0.04%– 37.596%) single HLA-C*07:02/IE-1^MAGE-^ multimer^+^ CD8^+^ T cells (Fig 3A). KIR-adjusted, truly HLA-C*07:02/IE-1-specific T cell frequencies were now reduced by 40.6%, but they still remained significantly higher with regard to HLA-B*07:02/pp65 multimer^+^ T cells (6.86% vs. 1.67%; p = 0.0294). Correlation of KIR-adjusted multimer^+^ and IFNγ^+^ CD8^+^ T cells revealed now an almost perfect fit of r^2^ = 0.96 (Fig 3B).

### Phenotypical analysis of HLA-C*07:02/IE-1^MAGE-^ and HLA-B*07:02/pp65 multimer^+^ T cells

**Fig 4:**
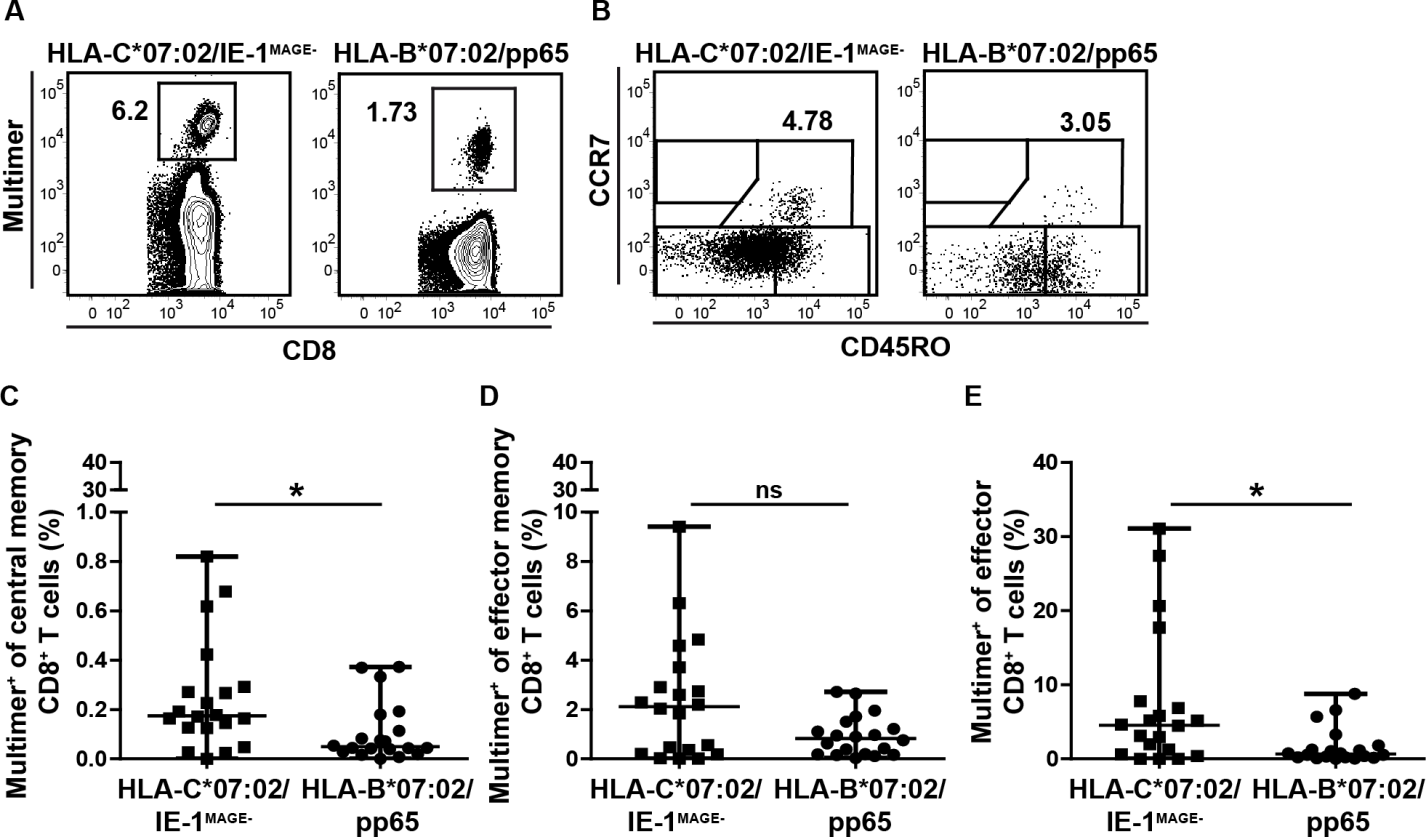
Phenotypic characterization of HLA-C*07:02/IE-1^MAGE-^ and HLA-B*07:02/pp65 multimer^+^ T cells. Phenotypic analysis of HLA-C*07:02/IE-1^MAGE-^- or HLA-B*07:02/pp65-specific CD8^+^ naïve (CCR7^+^CD45RO^-^), central memory (CCR7^+^CD45RO^+^), effector memory (CCR7^-^CD45RO^+^) and effector (CCR7^-^CD45RO^-^) T cells. **(A)** Exemplary multimer CD8^+^ T cell stainings and **(B)** differentiation phenotypes for the corresponding epitopes are shown. **(C)** Central memory, **(D)** effector memory and **(E)** effector CD8^+^ T cell subset frequencies (%) from HLA-C*07:02/ HLA-B*07:02 donors are compared for the respective epitopes. T cells were uniformly pre-gated on living CD3^+^/CD8^+^ lymphocytes. Statistical analyses were performed with the Mann-Whitney *U* test. * = p < 0.05, ** = p < 0.01, *** = p < 0.001.

With the ability to visualize KIR-adjusted HLA-C*07:02/IE-1-specific T cell populations by HLA-C multimer double-staining, we were interested in the distribution of their CD8^+^ T cell subsets characterized by the expression of CCR7 and CD45RO (T naïve (T_naive_) = CCR7^+^CD45RO^-^; T central memory (T_CM_) = CCR7^+^CD45RO^+^; T effector memory (T_EM_) = CCR7^-^CD45RO^+^; T effector (T_eff_) = CCR7^-^CD45RO^-^). In an exemplary donor with 6.2% HLA-C*07:02/IE-1^MAGE-^- and 1.73% HLA-B*07:02/pp65-restricted CMV-specific CD8^+^ T cells (Fig 4A) we detected with 4.78% (HLA-C*07:02/IE-1^MAGE-^) and 3.05% (HLA-B*07:02/pp65) comparable T_CM_ frequencies (Fig 4B). In consequence, the overall higher level of HLA-C*07:02/IE-1-restricted T cells together with a comparable memory T cell distribution pattern leads to a prominent contribution to the T_CM_ compartment. Indeed, analysis of the distribution of both specificities within the T_CM_ compartment of all donors (n = 20) confirmed significantly higher median frequencies of 0.17% (0%– 0.82%) for HLA-C*07:02/IE-1^MAGE-^ and 0.05% (0%– 0.37%) for HLA-B*07:02/pp65 multimer^+^ T cells (p = 0.019; Fig 4C) among T_CM_. In addition, we found within the T_EM_ and T_eff_ compartment median frequencies for HLA-C*07:02/IE-1^MAGE-^ multimer^+^ cells of 2.19% (0.01%– 9.41%) and 4.63% (0.01%– 31.08%), respectively. Frequencies for HLA-B*07:02/pp65 multimer^+^ cells were again lower, with T_EM_ frequencies of 0.82% (0.03%– 2.71%) and T_eff_ of 0.64% (0.02%– 8.79%), the latter in a significant manner (p = 0.096; p = 0.024; Fig 4D and 4E). The solid T_CM_ fraction among HLA-C*07:02/IE-1-specific T cells indicates a high proliferative capacity, which has been demonstrated previously to be relevant for *in vivo* expansion upon antigen re-encounter as well as for adoptive T cell therapy.

### Strong and functional expansion of HLA-C*07:02/IE-1-restricted T cells may contribute to virus-control in a CMV-reactivating kidney transplant recipient

**Fig 5:**
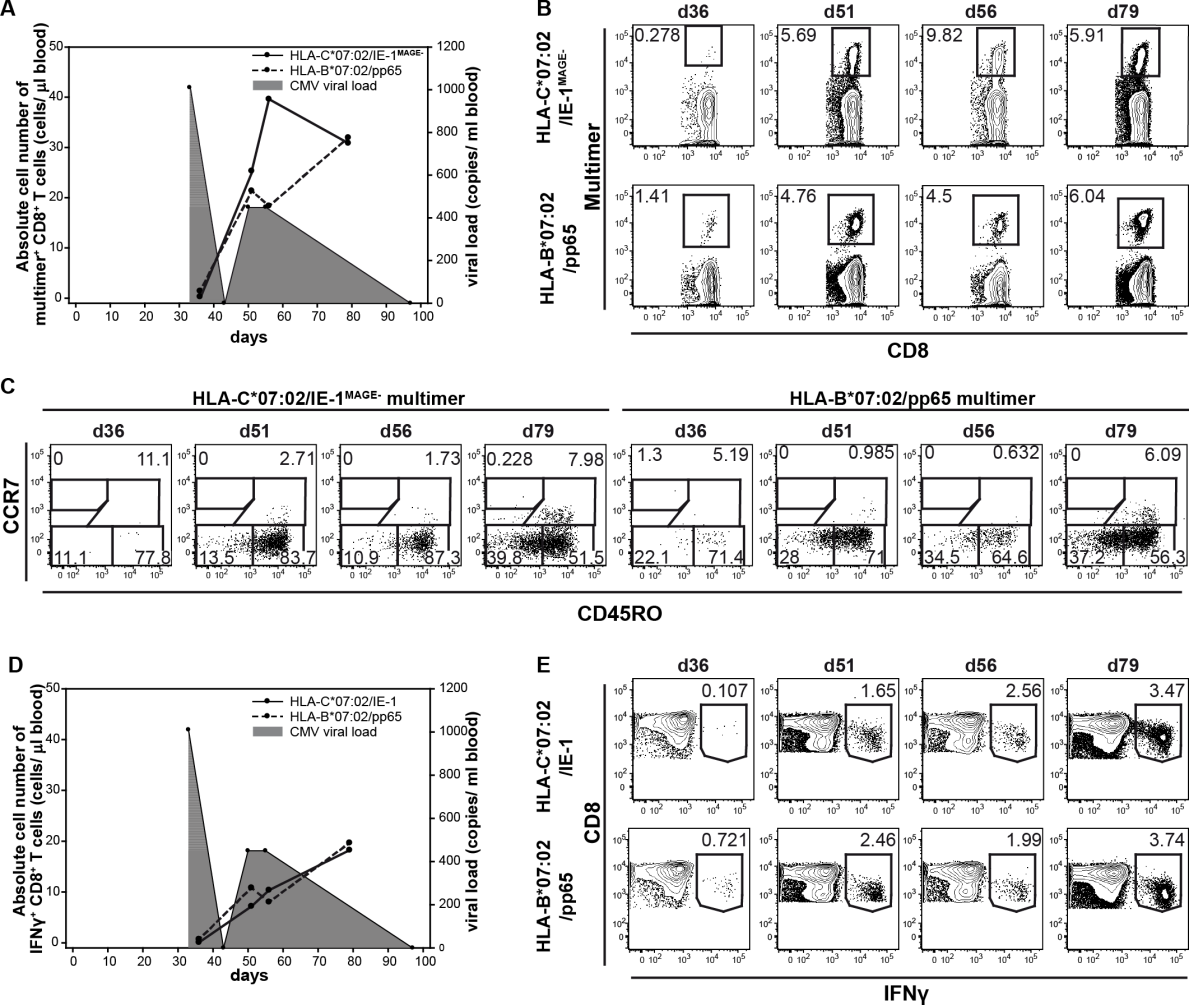
Comparable expansion kinetics of HLA-C*07:02/IE-1^MAGE-^- and HLA-B*07:02/pp65-specific T cells after CMV-reactivation of a kidney transplant recipient. Monitoring of expanding CMV-specific T cells in a kidney transplant recipient (D+/R+) with CMV-reactivation. **(A)** Kinetics of HLA-C*07:02/IE-1^MAGE-^- (solid line) and HLA-B*07:02/pp65 (dashed line)–multimer^+^ CD8^+^ T cells are shown. CMV-viremia (grey area) was measured by quantitative PCR. **(B)** Dot plots and **(C)** phenotypical analysis of expanding multimer^+^ CD8^+^ T cells for the corresponding epitopes are shown. **(D)** Kinetics of IFNγ^+^ CD8^+^ T cells after stimulation either with HLA-C*07:02/IE-1 peptide (solid line) or HLA-B*07:02/pp65 peptide (dashed line) followed by intracellular cytokine staining. CMV-viremia (grey area) is indicated. **(E)** Dot plots of expanding IFNγ^+^ CD8^+^ T cells for the corresponding epitopes are shown. T cells were pre-gated on living CD3^+^/CD8^+^ lymphocytes.

In order to analyze the expansion capacity and phenotype of HLA-C*07:02/IE-1-restricted T cells in a clinically relevant setting, we monitored a CMV-seropositive kidney transplant recipient with confirmed CMV-reactivation in the absence of antiviral prophylaxis. Both HLA-C*07:02/IE-1^MAGE-^ and HLA-B*07:02/pp65-specific T cells were in the beginning of CMV reactivation hardly detectable, which correlated with low absolute CD3^+^ T cell levels. Proliferation of CMV-specific CTLs for both epitopes was associated with decreased viral load, suggesting the establishment of protective immunity (Fig 5A). Interestingly, HLA-C*07:02/IE-1^MAGE-^-specific T cells showed an even more intense proliferation starting with 0.278% on day 36 and peak CTL levels of 9.82% on day 56, in parallel to viral control (Fig 5B). HLA-B*07:02/pp65-specific CD8^+^ T cells expanded in similar kinetics, but less intensively (Fig 5B). In a second step, we analyzed the phenotype of both specificities and were able to detect CMV-specific CD8^+^ central memory T cells (CCR7^+^/CD45RO^+^) for both specificities. After viral clearance, high numbers of virus-specific central memory T cells remained detectable, indicating the generation of a long lasting protective T cell memory (Fig 5C). In concordance with the MHC multimer results, we detected expanding levels of IFNγ-producing CD8^+^ T cells for both specificities (Fig 5D and 5E). This clinical example suggests a contribution of HLA-C*07:02/IE-1-specific T cells to the control of CMV reactivation and underlines the potential value of this population as a constituent of predictive diagnostic panels as well as a target for adoptive T cell therapy.

### Magnetic purification of HLA-C*07:02/IE-1-specific T cells

**Fig 6:**
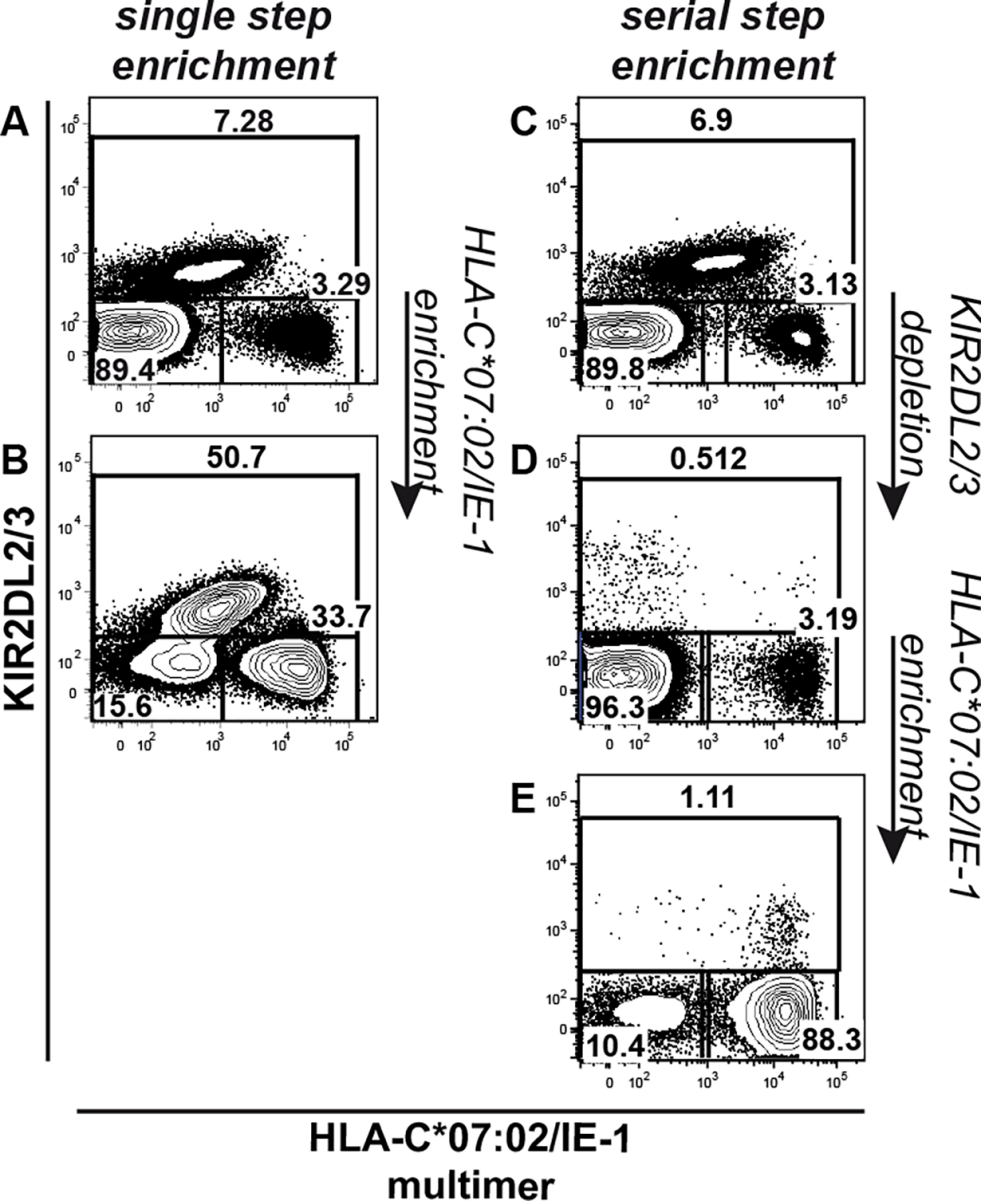
Magnetic purification of KIR-depleted HLA-C*07:02/IE-1-specific T cells. **(A-C)** Enrichment of KIR-adjusted HLA*07:02/IE-1-specific T cells was performed with an initial KIR2DL2/3 depletion step using KIR2DL2/3 PE-conjugated antibodies and anti-PE MicroBeads. KIR-negative cells were then positively selected with Microbead-coupled HLA-C*07:02/IE-1-Streptamers. **(A)** KIR2DL2/3 and HLA*07:02/IE-1-multimer stainings of unmanipulated donor T cells. **(B)** Depiction of the KIRDL2/3-depleted fraction and **(C)** of the final HLA-C*07:02/IE-1-multimer-enriched fraction are shown. Dot blots were uniformly pre-gated on living CD3^+^ lymphocytes.

TCR-independent, KIR2DL2/3-mediated enrichment during magnetic-bead-coupled HLA-C*07:02/IE-1 Streptamer purification could lead to a substantial contamination with potentially alloreactive T cells (Fig 6A and 6B). This would limit significantly the clinical use of this otherwise very interesting CMV-specific T cell product. Therefore, we conceived a serial magnetic enrichment protocol to isolate KIR2DL2/3-depleted, HLA-C*07:02/IE-1 Streptamer-purified T cells. In a proof-of-concept experiment we used magnetic-bead coupled, KIR2DL2/3-specific antibodies to deplete first KIR2DL2/3-expressing T cells. Prior to depletion, the donor had a frequency of 6.9% KIR2DL2/3^+^ cells and 3.13% of KIR-adjusted HLA-C*07:02/IE-1-restricted T cells (Fig 6C). The depletion of KIR-expressing cells was highly efficient with a reduction of 93%, which left only a negligible fraction of 0.512% KIR2DL2/3^+^ cells in the intermediate cell product (Fig 6D). Subsequently, we conducted a second, conventional positive enrichment step with magnetic bead-coupled HLA-C*07:02/IE-1 Streptamers. This led to a purity of 88.3% for HLA-C*07:02/IE-1-restricted T cells in the final T cell product (Fig 6E). This promising approach can be further developed to a GMP compatible process and should preserve, with the verified reversibility of HLA-C*07:02/IE-1 Streptamers (S4 Fig), the functional and regulatory advantages of minimally manipulated MHC Streptamer-purified T cell products.

## Discussion

CMV is still the most common viral infection after solid organ or stem cell transplantation [3, 4] and can be treated by adoptive transfer of CMV-specific T cells in the setting of HSCT [7–12]. Here we show that HLA-C*07:02/IE-1-specific T cells represent a large, functional and T_CM_-rich T cell population, which could play a beneficial role for optimized immune monitoring and adoptive T cell therapy.

Our analysis of donors with the HLA allele C*07:02, which is very common in Caucasians [24], indicated a broad availability of HLA-C*07:02/IE-1-specific T cells. After exclusion of epitope-independent binding via MHC multimer double staining they were found in high frequencies among CD8^+^ T cells containing stable fractions of early differentiation phenotypes. This observation underlines previous findings supporting an immunodominance of this epitope [20]. As there is a possible link between population size and enrichment efficiency in apheresis products from healthy donors [16], the prominence of HLA-C*07:02/IE-1-specific T cell populations could have a beneficial role for the processing of these cells with regard to purity and availability of sufficient CMV-specific T cells for adoptive T cell therapy.

The interaction of HLA-C*07:02 and KIR2DL2/3 prohibiting NK cell mediated killing [20–22] is potentially responsible for reduced immune evasion of HLA-C*07:02-expressing CMV-infected cells [20]. Interestingly, this well-known interaction of HLA-C1 receptors with KIR2DL2/3 [22, 28, 30] was obviously strong enough to allow epitope-independent staining with HLA-C*07:02 Streptamers. In previous analyses (data not shown) using MHC multimers containing HLA-A24, a member of the Bw4 group [28], we could not detect KIR (KIR3DL1)-associated binding. This indicates that specifically HLA-C*07:02- and potentially also other HLA-C multimers have a sufficiently strong binding capacity for KIR molecules expressed on T cells, which requires the adaptation of the well-established single-step magnetic MHC-enrichment protocols for the generation of primary antigen-specific CD8^+^ T cells [12, 16].

In the clinical setting of adoptive T cell transfer after allo-HSCT, depletion of KIR-expressing epitope- unspecific T cells is necessary to prevent enrichment of potentially alloreactive T cells, which could mediate graft-versus-host disease. As a depletion step with HLA-C*07:02/MAGE Streptamers was found to be technically challenging (data not shown), we used instead a KIR2DL3-specific antibody for depletion before the HLA-C*07:02/IE-1 Streptamer purification. This procedure worked sufficiently well, but for clinical applications a fully reversible Fab Streptamer for KIR2DL2/3 [31] would be desirable. This could prevent regulatory issues that would be raised with the transfer of not completely depleted KIR2DL3 antibody-coated cells. Fortunately, full reversibility of HLA-C*07:02/IE-1 Streptamers also needed in this context could be demonstrated by addition of D-biotin, as it has been shown earlier for HLA-A and HLA-B Streptamers (Knabel et al. 2002).

In addition, we were able to test the newly generated HLA-C Streptamer in a clinical monitoring setting. In several clinical trials the adaptive immunity in context of CMV has been analyzed by MHC tetramer staining and cytokine secretion assays and there is consensus that CMV-specific T cells play a major role in viral control. Phenotype, proliferation, absolute numbers and cytokine profiles are crucial for protection, as well as prediction of CMV-associated complications [18, 32–38]. Our newly developed combinatorial HLA-C multimer staining approach enables now the characterization of HLA-C*07:02/IE-1-specific T cells in a clinical setting. They showed strong proliferation and intense cytokine production after stimulation both being indicative for robust CMV immunity and qualifying them as an interesting target for the immune monitoring of transplanted patients. If, based on the stable expression of HLA-C7 on CMV-infected cells [20], HLA-C*07:02/IE-1-specific T cells have advantages in viral control still needs to be clinically elucidated, e.g. by the use of this T cell population in adoptive transfer trials.

Furthermore, we and others have described the beneficial role of early differentiated T cells in adoptive T cell therapy due to protracted survival and increased proliferation capacities [11, 14, 39, 40]. We could detect high numbers of central and effector memory T cells with HLA-C*07:02/IE-1-specificity, underlining the possible potential of this population. We recently initiated an interventional clinical trial in patients after HSCT in which recipients receive low doses of Fab Streptamer-selected central memory T cells (T_CM_) containing the complete CD4^+^ and CD8^+^ donor T cell repertoire (PACT, EudraCT-No. 2015-001522-41). A large majority of HLA-C*07:02-positive donor-derived T_CM_ products will be likely to comprise HLA-C*07:02/IE-1-specific T cells allowing precise clinical characterization of this novel T cell population in the context of prophylactic adoptive T cell transfer.

## Supporting information

S1 Fig Gating strategy for CMV-specific T cell staining. **(A)** Gating strategy for multimer staining. After selecting for living CD3^+^ CD8^+^ lymphocytes, multimer frequencies were assessed. If applicable, the differentiation phenotype of multimer^+^ T cells was analyzed. **(B)** Gating strategy for ICS. After selecting for living CD3^+^ IFNγ^+^ lymphocytes and CD8^+^ T cells, cytokine production was analyzed. 1x10^6^ PBMCs/ staining were used for both flow cytometric analyses.
(TIF)

S2 Fig Functional profile of HLA-C*07:02/IE-1- and HLA-B*07:02/pp65-specific CD8^+^ T cells. Analysis of T cell frequencies for functional HLA-C*07:02/IE-1- and HLA-B*07:02/pp65-restricted T cells. PBMCs from healthy donors (n = 20) were stimulated with corresponding epitopes and an ICS was performed. Shown are the frequencies of **(A)** CD8^+^ TNFα-producing T cells, **(B)** CD8^+^ IL-2-producing T cells, and **(C)** CD8^+^ GM-CSF-producing T cells. T cells were uniformly pre-gated on living CD3^+^/CD8^+^ lymphocytes. Statistical analyses were performed with the Mann-Whitney *U* test. * = p < 0.05, ** = p < 0.01, *** = p < 0.001.
(TIF)

S3 Fig CD107a-production of HLA-C*07:02/IE-1- and HLA-B*07:02/pp65-specific CD8+ T cells. **(A)** Representative intracellular CD107a staining of CD8+ T cells from a healthy donor restimulated with the corresponding epitopes. **(B)** Comparative T cell analysis of a group of healthy donors (n = 6) carrying both CMV-specific T cell populations. ICS of CD8^+^ CD107a-producing T cells after stimulation with corresponding epitopes. Plots were uniformly pre-gated on living CD3^+^/CD8^+^ lymphocytes. Statistical analyses were performed with the Mann-Whitney U test. * = p < 0.05, ** = p < 0.01, *** = p < 0.001.
(TIF)

S4 Fig Reversibility of HLA-C/IE-1-restricted Streptamers. PBMCs were stained by multimer double staining either before (left column) or after D-biotin treatment (middle left column). Residual MHC-monomers were then analyzed by restaining with StrepTactin APC (middle right column). Secondary MHC-multimer staining served as a control (right column). T cells were uniformly pre-gated on living CD3^+^/CD8^+^ lymphocytes.
(TIF)
