# Supplementary figures and images for: Characterization and clinical enrichment of HLA-C*07:02-restricted Cytomegalovirus-specific CD8+ T cells
Source: PLoS One. 2018 Feb 28;13(2):e0193554. doi: 10.1371/journal.pone.0193554 (PMC5831000; doi:10.1371/journal.pone.0193554)

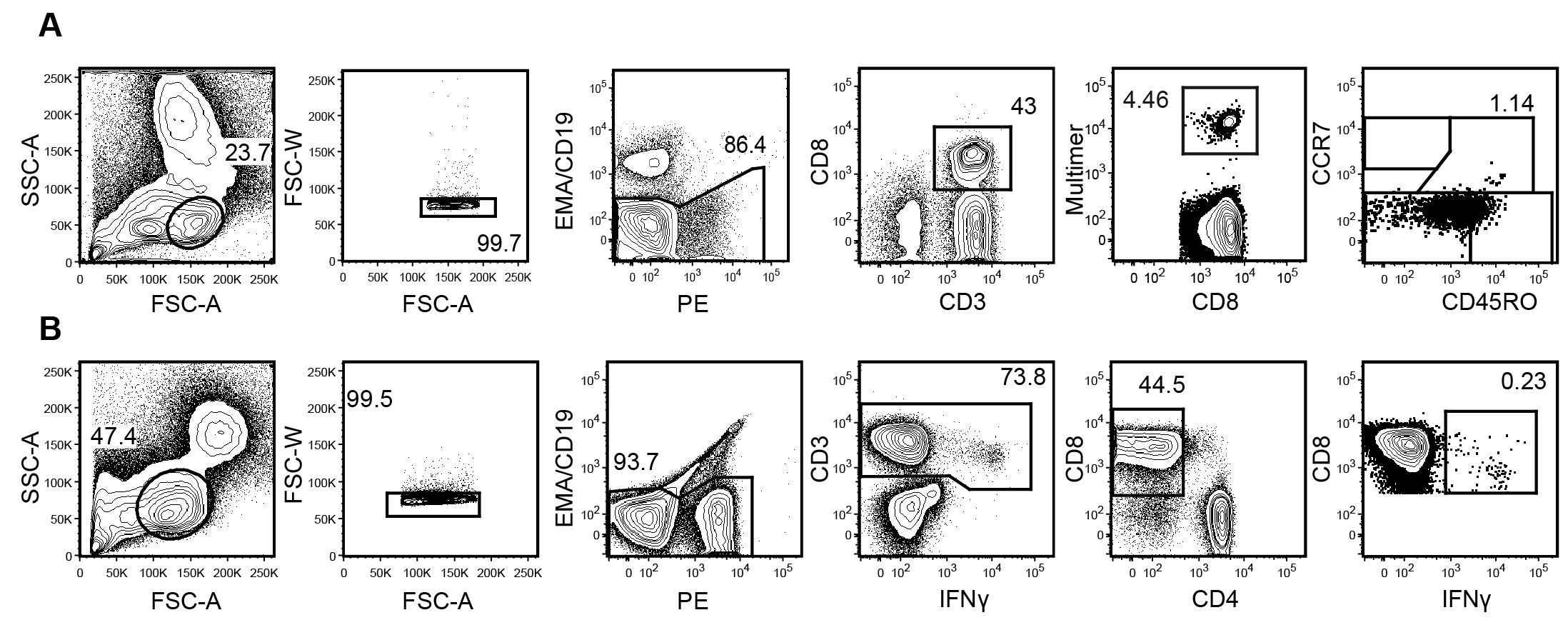

Supplement: S1 Fig — (A) Gating strategy for multimer staining. After selecting for living CD3+ CD8+ lymphocytes, multimer frequencies were assessed. If applicable, the differentiation phenotype of multimer+ T cells was analyzed. (B) Gating strategy for ICS. After selecting for living CD3+ IFNγ+ lymphocytes and CD8+ T cells, cytokine production was analyzed. 1x10^6^ PBMCs/ staining were used for both flow cytometric analyses. (TIF) [file pone.0193554.s001.tif]

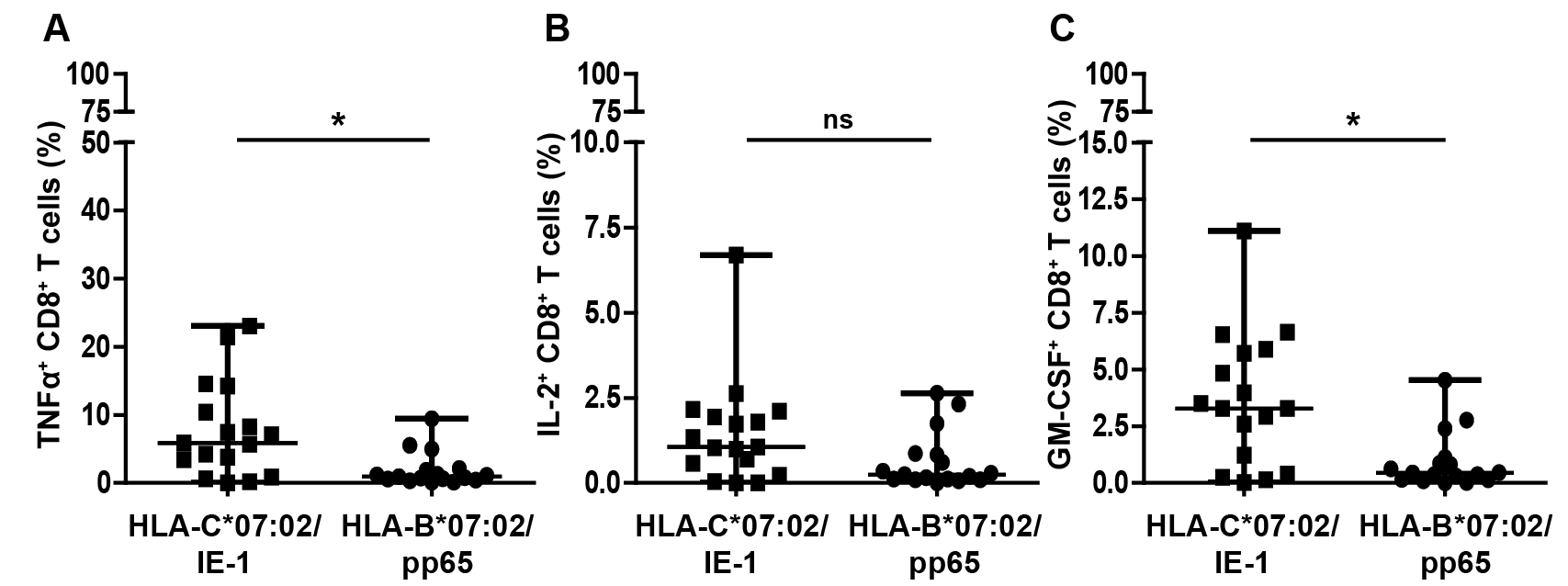

Supplement: S2 Fig — Analysis of T cell frequencies for functional HLA-C*07:02/IE-1- and HLA-B*07:02/pp65-restricted T cells. PBMCs from healthy donors (n = 20) were stimulated with corresponding epitopes and an ICS was performed. Shown are the frequencies of (A) CD8+ TNFα-producing T cells, (B) CD8+ IL-2-producing T cells, and (C) CD8+ GM-CSF-producing T cells. T cells were uniformly pre-gated on living CD3+/CD8+ lymphocytes. Statistical analyses were performed with the Mann-Whitney U test. * = p < 0.05, ** = p < 0.01, *** = p < 0.001. (TIF) [file pone.0193554.s002.tif]

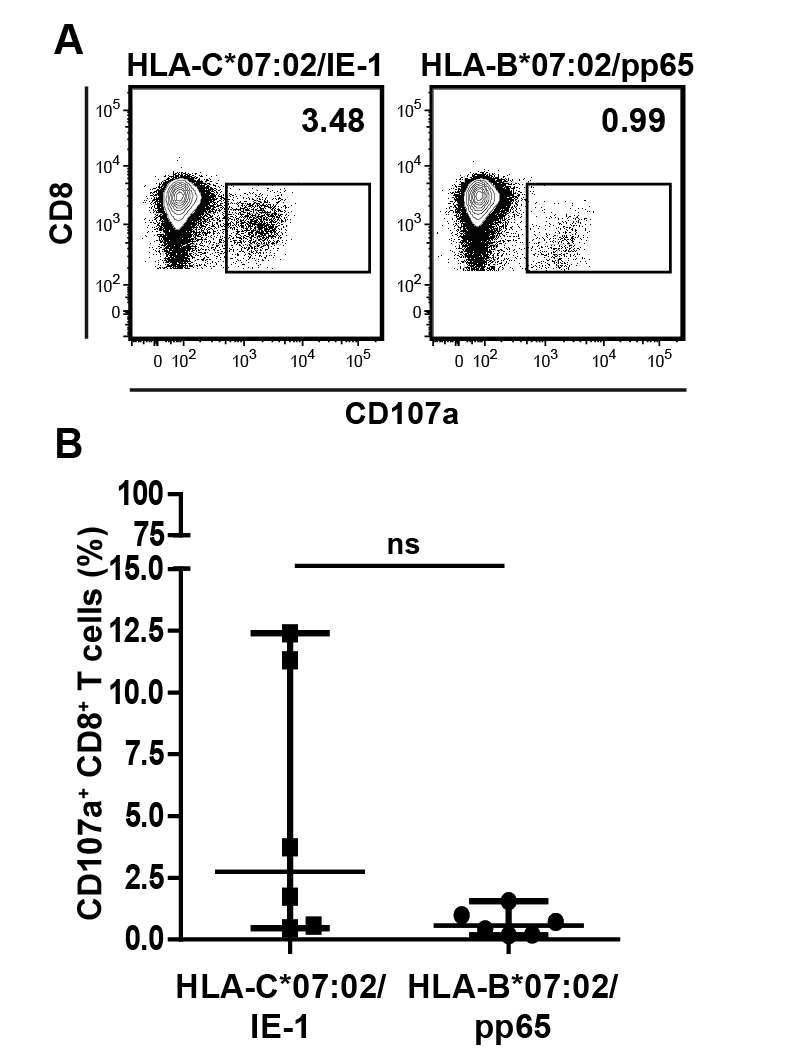

Supplement: S3 Fig — (A) Representative intracellular CD107a staining of CD8+ T cells from a healthy donor restimulated with the corresponding epitopes. (B) Comparative T cell analysis of a group of healthy donors (n = 6) carrying both CMV-specific T cell populations. ICS of CD8+ CD107a-producing T cells after stimulation with corresponding epitopes. Plots were uniformly pre-gated on living CD3+/CD8+ lymphocytes. Statistical analyses were performed with the Mann-Whitney U test. * = p < 0.05, ** = p < 0.01, *** = p < 0.001. (TIF) [file pone.0193554.s003.tif]

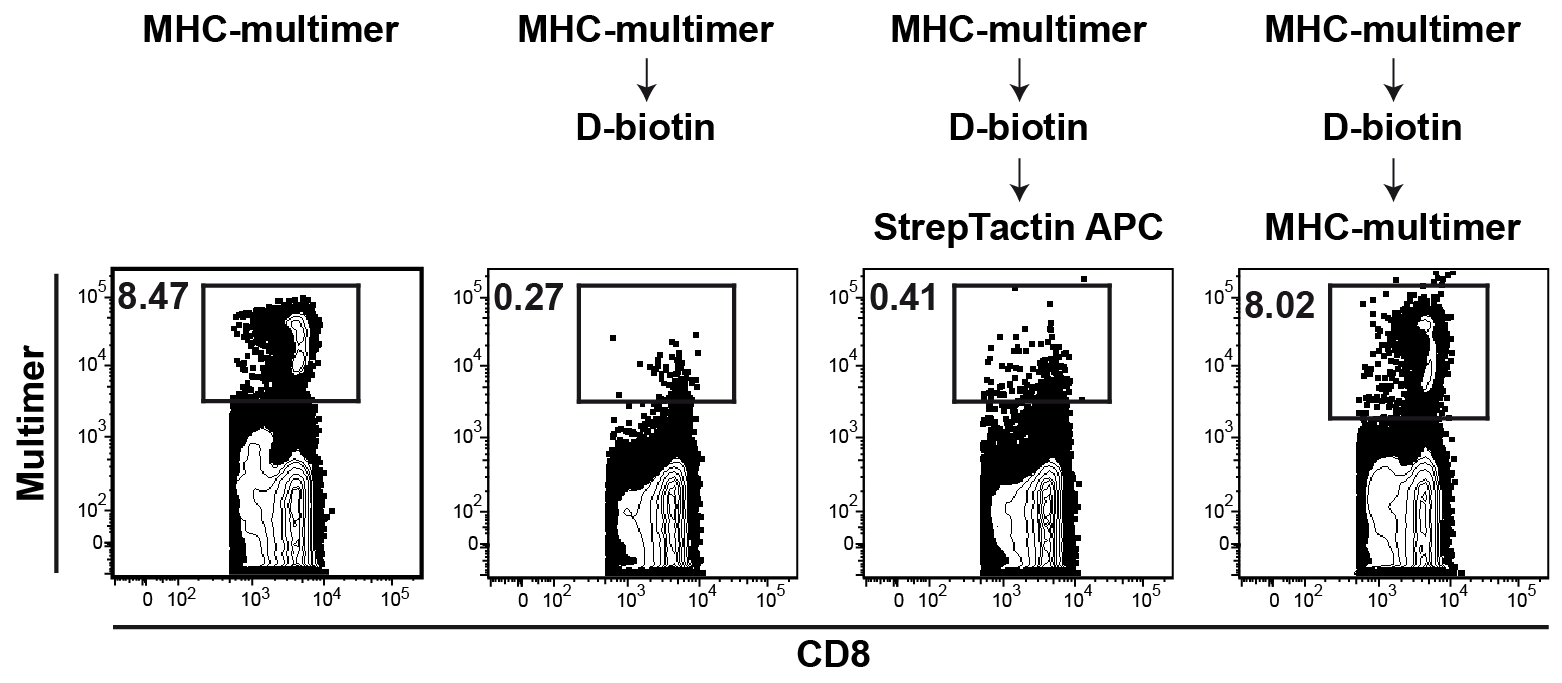

Supplement: S4 Fig — PBMCs were stained by multimer double staining either before (left column) or after D-biotin treatment (middle left column). Residual MHC-monomers were then analyzed by restaining with StrepTactin APC (middle right column). Secondary MHC-multimer staining served as a control (right column). T cells were uniformly pre-gated on living CD3+/CD8+ lymphocytes. (TIF) [file pone.0193554.s004.tif]
